# 3D architecture and structural flexibility revealed in the subfamily of large glutamate dehydrogenases by a mycobacterial enzyme

**DOI:** 10.1038/s42003-021-02222-x

**Published:** 2021-06-03

**Authors:** Melisa Lázaro, Roberto Melero, Charlotte Huet, Jorge P. López-Alonso, Sandra Delgado, Alexandra Dodu, Eduardo M. Bruch, Luciano A. Abriata, Pedro M. Alzari, Mikel Valle, María-Natalia Lisa

**Affiliations:** 1 Center for Cooperative Research in Biosciences (CIC bioGUNE), Basque Research and Technology Alliance (BRTA), Bizkaia Technology Park, Building 801 A, Derio, Spain; 2 Centro Nacional de Biotecnología, CNB-CSIC, Darwin 3, Madrid, Spain; 3 Unité de Microbiologie Structurale, Institut Pasteur, CNRS UMR 3528, Université de Paris, 25 rue du Docteur Roux, Paris, France; 4 Integrated Structural Biology Department, IGBMC, 1 Rue Laurent Fries, Illkirch, France; 5 Laboratory for Biomolecular Modeling, School of Life Sciences, École Polytechnique Fédérale de Lausanne and Swiss Institute of Bioinformatics, Lausanne, Switzerland; 6 Protein Production and Structure Core Facility, School of Life Sciences, École Polytechnique Fédérale de Lausanne, Lausanne, Switzerland; 7 Instituto de Biología Molecular y Celular de Rosario (IBR, CONICET-UNR), Ocampo y Esmeralda, Rosario, Argentina; 8 Plataforma de Biología Estructural y Metabolómica (PLABEM), Ocampo y Esmeralda, Rosario, Argentina; 9 Present Address: CH, DBV-Technologies, 177-181 Avenue Pierre Brossolette, 92120 Montrouge, France; EMB, Unité de Microbiologie Structurale, Institut Pasteur, CNRS UMR 3528, Université de Paris, 25 rue du Docteur Roux, Paris, France

**Keywords:** X-ray crystallography, Cryoelectron microscopy, Pathogens

## Abstract

Glutamate dehydrogenases (GDHs) are widespread metabolic enzymes that play key roles in nitrogen homeostasis. Large glutamate dehydrogenases composed of 180 kDa subunits (L-GDHs_180_) contain long N- and C-terminal segments flanking the catalytic core. Despite the relevance of L-GDHs_180_ in bacterial physiology, the lack of structural data for these enzymes has limited the progress of functional studies. Here we show that the mycobacterial L-GDH_180_ (mL-GDH_180_) adopts a quaternary structure that is radically different from that of related low molecular weight enzymes. Intersubunit contacts in mL-GDH_180_ involve a C-terminal domain that we propose as a new fold and a flexible N-terminal segment comprising ACT-like and PAS-type domains that could act as metabolic sensors for allosteric regulation. These findings uncover unique aspects of the structure-function relationship in the subfamily of L-GDHs.

## Abstract (web summary)

Lázaro *et. al*. report the first 3D structure of a large glutamate dehydrogenase (L-GDH), the one corresponding to the *Mycobacterium smegmatis* enzyme composed of 180 kDa subunits (mL-GDH_180_), obtained by X-ray crystallography and cryo-electron microscopy. This structure reveals that mL-GDH_180_ assembles as tetramers with the N- and C-terminal domains being involved in inter-subunit contacts and unveils unique features of the subfamily of L-GDHs.

## Introduction

Glutamate dehydrogenases (GDHs) are ubiquitous oligomeric enzymes that catalyze the reversible oxidative deamination of l-glutamate to 2-oxoglutarate, at the crossroad between the Krebs cycle and ammonium assimilation. GDHs are grouped into the subfamily of small GDHs composed of subunits of 50 kDa (S-GDHs_50_) and the subfamily of large GDHs (L-GDHs) composed of monomers of 115 kDa (L-GDHs_115_) or 180 kDa (L-GDHs_180_)^1^. L-GDHs, found in lower eukaryotes and prokaryotes, are NAD^+^ dependent enzymes that differ from S-GDHs_50_ by the presence of long N- and C-terminal extensions flanking the catalytic domain^1^. The possible role(s) of such terminal segments in oligomerization and/or enzyme regulation has remained largely unknown^1–9^.

The relevance of L-GDHs_180_ in bacterial physiology has been emphasized in previous studies of environmental^8^ and pathogenic species^10,11^. Among the later, the mycobacterial L-GDH_180_ (mL-GDH_180_) is part of a signal transduction pathway that senses amino acid availability to control metabolism and virulence of *Mycobacterium tuberculosis*^7,12,13^. This enzyme is essential for the in vitro growth of the tubercle bacillus^10,11^ whereas it is crucial for *Mycobacterium bovis* BCG survival in media containing glutamate as the sole carbon source^14^. Moreover, diverse mechanisms have been implicated in the regulation of L-GDHs_180_. The catabolism of glutamate by mL-GDH_180_ is inhibited by the regulator GarA^6,7^ when extracellular nitrogen donor amino acids are available^12^ whereas the L-GDH_180_ from *Streptomyces clavuligerus*^1^ (filo Actinobacteria, which includes mycobacteria) as well as L-GDHs_180_ from Proteobacteria^2,4,5^ are directly regulated by amino acids. Despite the key roles of L-GDHs_180_ in the redistribution of amino groups within cells, their 3D structure has remained elusive, preventing a deeper understanding of the molecular basis of enzyme function.

Here we report the 3D structure of the mL-GDH_180_ isoform from *Mycobacterium smegmatis*, obtained through an integrative approach that combined single-particle cryo-EM and X-ray protein crystallography data at resolutions between 3.59 and 6.27 Å. Our findings reveal unique characteristics of domain organization and oligomeric assembly in the L-GDHs subfamily, thus allowing to update the annotation of the Pfam family PF05088 that includes the L-GDHs_180_, and offer a rationale for the direct regulation of L-GDHs_180_ by metabolites. Furthermore, our cryo-EM data uncover fluctuations of the quaternary structure of mL-GDH_180_ that are possibly relevant for the allosteric regulation of the enzyme activity.

## Results

### The 3D architecture of mL-GDH_180_

**Fig. 1:**
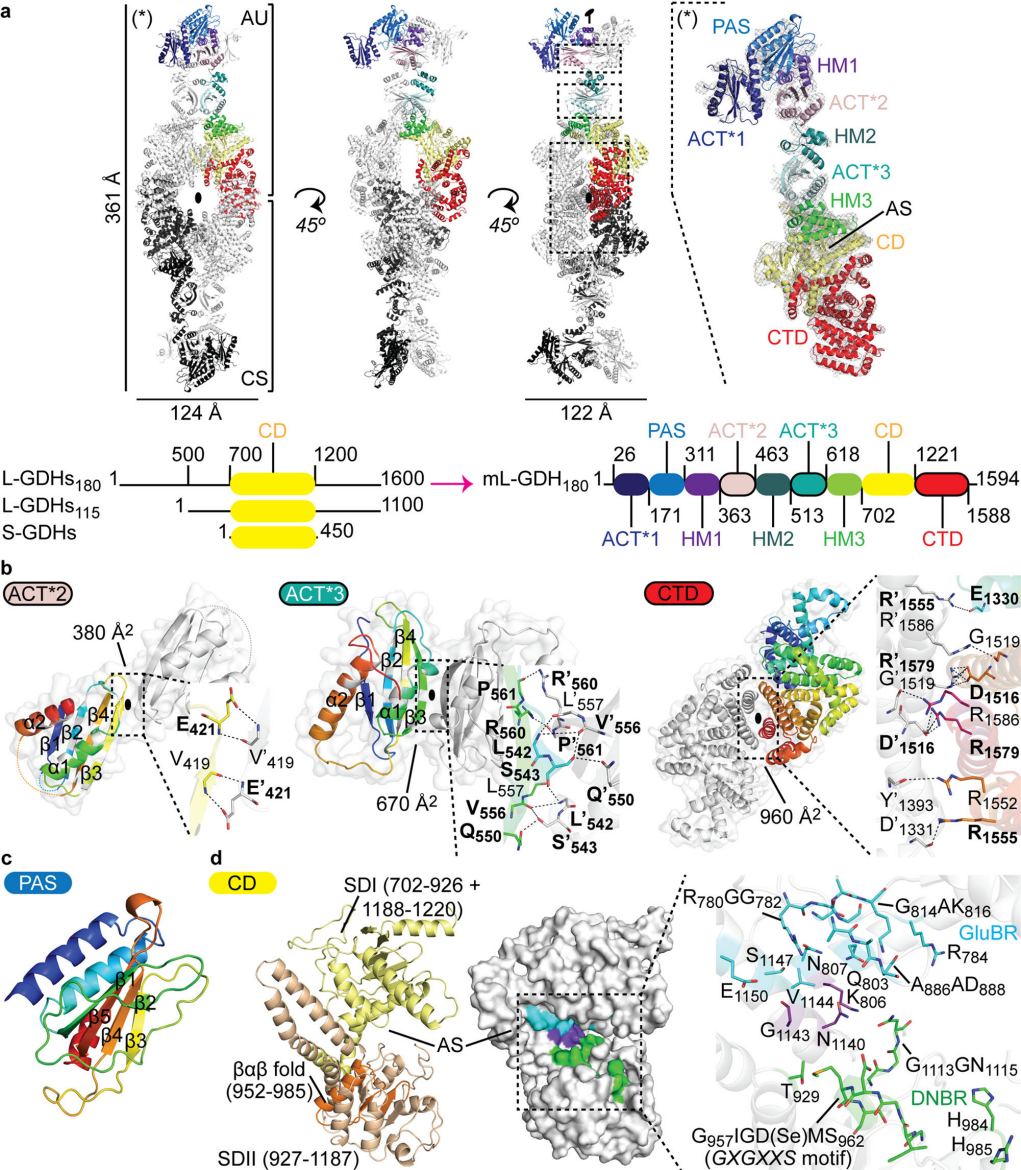
Crystal structure of Se-Met mL-GDH180. **a** The asymmetric unit (AU) contains two monomers (RMSD of 0.51 Å for 289 alpha carbons in segment 45-362, 0.26 Å for 1163 alpha carbons in segment 368-1588); a tetramer (as ribbons) is formed by crystallographic symmetry (CS); oval symbols represent twofold axes. The *2mFo–DFc* electron density (gray mesh), contoured to 1.5σ, is shown for one protein subunit on the right. Domains boundaries are given in residue numbers in a scheme below; CD catalytic domain, CTD C-terminal domain, AS active site. A comparative scheme of L-GDHs_180_, L-GDHs_115_, and S-GDHs_50_ is also provided, with approximate residue numbers. **b** Oligomeric interfaces (areas in Å^2^) involve the domains ACT*2, ACT*3, and CTD. Contacting residues (as sticks in insets) labeled in bold characters are strictly conserved in diverse L-GDHs. The topology of domains ACT*2 and ACT*3 is highlighted with rainbow colors; white positions within the rainbow depict conserved core residues^15^. **c** The PAS domain. **d** The CD is shown with the SDI and SDII in yellow and orange, respectively. The βαβ motif is involved in dinucleotide binding^1^. The glutamate-binding region (GluBR, cyan) and the dinucleotide-binding region (DNBR, green)^1^ are highlighted in a surface representation and as sticks in an inset. Residues in purple conform both binding regions 1. See also Supplementary Figs. 1 and 2.

**Fig. 2:**
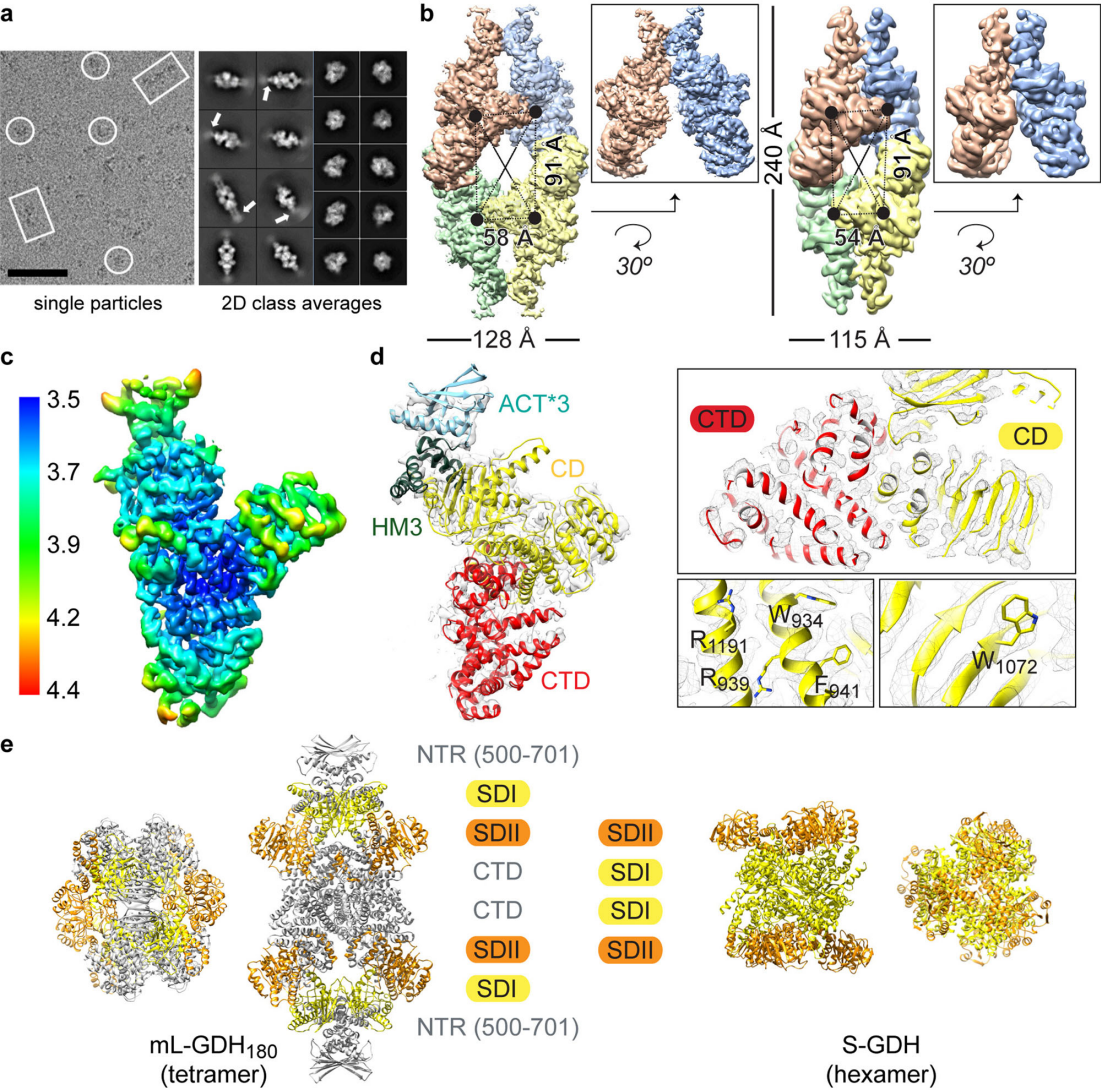
Intrinsic flexibility and alternate conformers of native mL-GDH180. **a** Cryo-EM image obtained for mL-GDH_180_ showing side (rectangles) and top (circle) views for single particles. Scale bar: 50 nm. The 2D class averages for mL-GDH_180_ tetramers display flexible ends at side views (white arrows). **b** Cryo-EM density maps for the open (left, 4.19 Å resolution) and closed (right, 6.6 Å resolution) conformations of mL-GDH_180_ tetramers, segmented into the four subunits. Black dots: centers of mass of the subunits. Insets are close-up views of the contact zone between the N-terminal regions (NTRs) of two monomers. **c** Local resolution for a single subunit of the open conformation after focused refinement (average resolution is 3.59 Å). **d** Cryo-EM map for one mL-GDH_180_ subunit and the fitted atomic coordinates. Domains colors and labels are as in Fig. 1. Insets are close-up views; selected amino acid side chains are shown as sticks. **e** Comparison of the quaternary structure of mL-GDH_180_, with D2 symmetry, and a representative hexameric S-GDH_50_ (PDB code 3SBO), with D3 symmetry. The catalytic domains are colored into SDI (yellow) and SDII (orange). The NTRs (only the portion that is well defined in cryo-EM maps is displayed) and the CTDs of mL-GDH_180_ monomers are depicted in gray. See also Supplementary Figs. 3 and 4.

As revealed by X-ray protein crystallography and single-particle cryo-EM (Figs. 1 and 2), mL-GDH_180_ assembles into a homotetramer. mL-GDH_180_ monomers are arranged around perpendicular twofold axes that pass through a central cavity in the structure.

The 6.27 Å resolution crystal structure of the seleno-methionine (Se-Met) derivative of mL-GDH_180_ (Fig. 1 and Table 1), obtained as illustrated in Supplementary Fig. 1 through an integrative strategy that also included cryo-EM data up to 3.59 Å, revealed that the protein subunits display, to the best of our knowledge, a unique domain organization (Fig. 1a). The N-terminal segment comprises three ACT (Aspartate kinase-Chorismate mutase-TyrA) -like^15^ (hereafter ACT*, see below) domains (ACT*1-3), a PAS (Per-Arnt-Sim) -type^16^ domain and three helical motifs (HM1-3). Notably, the primary structures of ACT and PAS domains are poorly conserved and, therefore, these modules are often difficult to identify from BLAST searches^15,16^. The C-terminal region consists of a single helical domain that showed no detectable structural similarity to previously characterized proteins in Dali^17^, ECOD^18^, CATH^19^, and VAST^20^ searches and, therefore, constitutes a possible new fold.

**Table 1:** X-ray diffraction data collection and refinement statistics.

	Se-Met mL-GDH_180_(PDB code 7JSR)
Data collection	
Space group	C222_1_
Cell dimensions	
* a*, *b*, *c* (Å)	151.6, 253.5, 399.7
*α*, *β*, *γ* (°)	90, 90, 90
Resolution (Å)	6.27-24.98 (6.27-7.01)^a^
* R*_merge_	0.055 (0.778)
* I* / σ*I*	10.4 (1.0)
CC (1/2)	0.999 (0.867)
Completeness (%)	98.4 (100)
Redundancy	4.9 (4.9)
Refinement	
Resolution (Å)	6.27-24.98
No. reflections	16,927
* R*_work_ / *R*_free_	27.7/32.5
No. atoms	
Protein	22,997
* B*-factors	
Protein	472.38
R.m.s. deviations	
Bond lengths (Å)	0.002
Bond angles (°)	0.63
Ramachandran	
Favored	93%
Allowed	6.8%
Outliers	0.2%

^a^One protein crystal was employed for structure determination. Values in parentheses are for highest-resolution shell.

The catalytic domains in the mL-GDH_180_ complex were not found to contribute intersubunit contacts (Fig. 1a). Instead, the N- and C-terminal regions of mL-GDH_180_ provide dimer-like interactions between pairs of monomers. Contacts between mL-GDH_180_ subunits engage the ACT*2, ACT*3, and C-terminal domains (Fig. 1b). Most of the residues involved in interfacial hydrogen bonds or salt bridges in mL-GDH_180_ are strictly conserved in the enzyme isoform from *M. tuberculosis* (O53203, 72% sequence identity)^7^, the L-GDH_180_ from *S. clavuligerus* (E2Q5C0, 47% sequence identity)^1^ and the L-GDH_115_ from *Nocardia farcinica* (A0A0H5NTF9, 55% sequence identity over non-gap aligned columns). Except for a single amino acid (Arg560), the same group of residues is also conserved in the L-GDH_180_ from *P. aeruginosa* (Q9HZE0, 40% sequence identity)^2^. These observations underscore the functional relevance of the oligomeric assembly found for mL-GDH_180_.

ACT and PAS modules are known to regulate functionally diverse proteins by driving conformational and/or quaternary structural changes^15,16^. The binding of specific amino acids to ACT–ACT interfaces confers allosteric control to oligomeric enzymes involved in amino acid metabolism^15^ whereas PAS modules sense and transduce chemical or physical stimuli to typically dimeric effector domains^16^. The ACT* domains of mL-GDH_180_ differ from the archetypal ACT fold in that strand β_1_ is located in the position usually occupied by strand β_4_, creating an ACT-like ββαββα topology with a β_1_β_2_β_4_β_3_ antiparallel sheet (Fig. 1b and Supplementary Fig. 2). Similar variations of the characteristic ACT fold have been described for aspartate kinases and a mammalian tyrosine hydroxylase^15,21^, including sixteen core residues that are conserved in the ACT*1-3 domains of mL-GDH_180_ (Supplementary Fig. 2). Notably, the interaction between ACT*3 modules in mL-GDH_180_ produces a continuous eight-stranded antiparallel β-sheet with helices on one side (Fig. 1b). A similar side-by-side arrangement of ACT domains generates allosteric amino acid binding sites in 3-phosphoglycerate synthases and aspartate kinases^15^. Close to a dimer-like interface, the PAS module in mL-GDH_180_ adopts a typical fold (Fig. 1c), comprising a core five-stranded β-sheet usually involved in signal sensing^16^, and displays up to 12% sequence identity with PAS domains in sensor histidine kinases retrieved in Dali^17^ searches.

Similarly to S-GDHs_50_, the catalytic core of mL-GDH_180_ consists of subdomains SDI and SDII (Fig. 1d), with the active site located in a groove in-between. Functionally important residues in the catalytic domain of L-GDHs_180_ have been previously identified by their conservation in sequence comparisons of diverse GDHs^1^. The SDI in mL-GDH_180_ contains most of the residues of the glutamate-binding region whereas the SDII conforms the dinucleotide-binding site.

### Intrinsic flexibility and alternate conformers of mL-GDH_180_

Cryo-EM and SAXS data uncovered the intrinsic flexibility of native mL-GDH_180_ (Fig. 2, Supplementary Fig. 3, and Supplementary Table 1). The domains comprised in the 1–500 region of the protein are stabilized by crystal contacts in the crystallographic structure. In contrast, 2D averages for side views of mL-GDH_180_ tetramers revealed a high degree of flexibility at distal ends, where ACT*1-2 and PAS domains reside, and their corresponding densities vanished in 3D cryo-EM maps (Fig. 2a). A 3D-classification of the detected mL-GDH_180_ particles was performed to distinguish alternate conformers of the enzyme. Two mL-GDH_180_ conformers were found, called the open and closed conformations (Fig. 2b), for which the ACT*3 module, the HM3, the catalytic domain and the C-terminal region were defined in each monomer, achieving an estimated 3.59 Å resolution for this region in the open conformation (Fig. 2c, d, Supplementary Figs. 4 and 5, and Table 2). The two conformers differ in the relative positions of the centers of mass of the subunits (Fig. 2b). The catalytic domains in mL-GDH_180_ monomers in contact through their N-terminal segments are found closer to each other in the less stable closed conformation compared to the open form. Overall, these findings reveal transitions of the quaternary structure that could intervene in the allosteric regulation of the enzyme.

**Table 2:** Cryo-EM data collection and processing.

	Open form (EMD-11606)	Closed form(EMD-11612)	Monomer(EMD-11613)
Data collection and processing			
Magnification	47,170	47,170	47,170
Voltage (kV)	300	300	300
Electron dose (e^*-*^/Å^2^)	40 (20 fractions)	40 (20 fractions)	40 (20 fractions)
Defocus range (μm)	0.67-3.26	0.67-3.26	0.67-3.26
Pixel size (Å)	1.06	1.06	1.06
Symmetry imposed	D2	D2	C1
Initial particles images (no.)	276,704	276,704	276,704
Final particles images (no.)	63,715	42,476	63,715 ×4
Map resolution (Å)	4.19	6.6	3.59
FSC threshold	0.143	0.143	0.143
Map sharpening B factor	−118.9	−250	−118.9
Refinement			
Initial model used (PDB code)	7JSR	–	–
Model resolution (Å)	4.03	–	–
FSC threshold	0.143	–	
Map sharpening B factor (Å^2^)	−118.9	–	–
Model composition			
Non-hydrogen atoms	33,032	–	–
Protein residues	4,252	–	–
Ligands	0	–	–
B factors (Å^2^)			
Protein	116.28	–	–
Ligand	–	–	–
R.m.s. deviations			
Bond lengths (Å)	0.006	–	–
Bond angles (°)	1.026	–	–
Validation			
MolProbity score	1.93	–	–
Clashscore	7.87	–	–
Poor rotamers (%)	0.35	–	–
Ramachandran plot			
Favored (%)	91.6	–	–
Allowed (%)	8.4	–	–
Disallowed (%)	0	–	–

## Discussion

L-GDHs_180_ were discovered in 2000 from a study of *Streptomyces clavurigerus*^1^ and were later isolated from other diverse bacterial species, including *Pseudomonas aeruginosa*^2^, psychrophilic bacteria^3–5^, *Caulobacter crescentus*^8^, and *Mycobacterium* spp^6,7^. As sequences of L-GDHs were identified, they were analyzed in light of the available crystallographic evidence for S-GDHs_50_^1,22^.

S-GDHs_50_ are hexameric enzymes in which the oligomeric interfaces are conformed by motifs that are located within the catalytic domain^1,22^. Most of these motifs are substantially modified in L-GDHs, either through sequence changes, insertions, or deletions^1,22^. In agreement with proposals that the oligomeric assembly would then be different for the two enzyme subfamilies^1,22^, the quaternary structure of mL-GDH_180_ depends on interactions established by the N- and C-terminal regions flanking the catalytic domain (Fig. 1a) and is radically different from that of S-GDHs_50_ (Fig. 2e). The stoichiometry of the mL-GDH_180_ complex observed by cryo-EM and X-ray protein crystallography (Figs. 1 and 2) is supported by molecular weight estimates from SAXS data (Supplementary Table 1) and is consistent with previous reports of tetrameric complexes of L-GDHs studied in solution^2,9^. Furthermore, most of the residues involved in interactions between mL-GDH_180_ monomers are conserved (Fig. 1b) not only in mycobacterial isoforms of the enzyme but also in L-GDHs from diverse species in Actinobacteria and Proteobacteria. Notably, the C-terminal domain, for which we did not find structural similarity with other characterized proteins, has a conserved length among L-GDHs (Fig. 1a) and the contacts between residues predicted from sequence alignments by Raptor X (Supplementary Fig. 6) further support the interactions observed experimentally. All these findings suggest that the oligomeric assembly of mL-GDH_180_ may be a common theme in the enzyme subfamily.

The catalytic domains in the mL-GDH_180_ complex are oriented opposite to those in S-GDHs_50_ (Fig. 2e), with the SDI (Fig. 1d) directed toward the distal ends of the protein, where the monomers N-terminal region resides. This segment comprises ACT-like modules as well as a PAS-like domain arranged in tandem (Fig. 1a) and shows a high degree of flexibility (Fig. 2a). A comparison of the mL-GDH_180_ conformers identified by cryo-EM (Fig. 2b) shows that conformational changes in the N-terminal region correlate with alterations in the relative positions of the catalytic domains. Taking into account the known roles of ACT modules in the allosteric control of oligomeric enzymes involved in amino acid metabolism^15^, our findings offer a rationale for previous evidence pointing out the direct regulation of diverse L-GDHs_180_ by metabolites^1,2^.

In conclusion, our findings suggest that the N-terminal segment of mL-GDH_180_ (as well as in related enzymes) could transduce intracellular metabolic stimuli to the catalytic core by driving changes in the quaternary structure. The reported 3D model of mL-GDH_180_ can now frame future studies to dissect the structure-function relationship of this enzyme and other members of the L-GDHs subfamily.

## Methods

### Protein production and purification

The sequence coding for the L-GDH_180_ from *M. smegmatis* MC^2^-155 (MSMEG_4699, Uniprot A0R1C2) was cloned into vector pLIC-His^23^ employing the oligonucleotides Fw: CCAGGGAGCAGCCTCGATGATTCGCCGGCTTTCGG and Rv: GCAAAGCACCGGCCTCGTTACCCAGTCGTTCCGGTCCC. The resulting plasmid was used to produce N-terminally His6-tagged mL-GDH_180_ in *E. coli* cells. Transformed *E. coli* cells were grown at 37 °C in medium supplemented with ampicillin or carbenicillin until reaching 0.8 units of optical density at 600 nm. Protein expression was then induced by adding isopropyl β-d-1-thiogalactopyranoside (IPTG) to a final concentration of 0.5 mM, and the incubation was continued for 18 h at 14 °C. Cells were harvested by centrifugation and sonicated. Following clarification by centrifugation, the supernatant was loaded onto a HisTrap HP column (GE Healthcare) equilibrated with buffer 25 mM HEPES, 500 mM NaCl, 20% v/v glycerol, 20 mM imidazole, pH 8.0, and His6-tagged mL-GDH_180_ was purified by applying a linear imidazole gradient (20–500 mM). The protein was then further purified by size-exclusion chromatography, as described below. mL-GDH_180_ containing fractions, as confirmed by SDS-PAGE and measurements of glutamate dehydrogenase activity^6^, were pooled and used immediately. The protein was quantified by electronic absorption using the molar absorption coefficient of 171,090 M^−1^ cm^−1^, predicted from the amino acid sequence by the ProtParam tool (http://web.expasy.org/protparam/).

For EM and SAXS experiments, native mL-GDH_180_ was produced in *E. coli* BL21(DE3) cells grown in LB broth. Size-exclusion chromatography was performed using a Superose 6 10/300 GL column (GE Healthcare) equilibrated in buffer 20 mM MES, 300 mM NaCl, 5 mM MgCl_2_, pH 6.0. Instead, Se-Met mL-GDH_180_ for crystallographic studies was produced in *E. coli* B834 (DE3) cells grown in SelenoMethionine Medium Complete (Molecular Dimensions), and size-exclusion chromatography was carried out using a HiPrep Sephacryl S-400 HR column (GE Healthcare) equilibrated in buffer 25 mM Tris, 150 mM NaCl, pH 7.5.

GarA from *M. tuberculosis* was produced as described by England et al.^24^.

### Cryo-electron microscopy

In all, 4 µl of 0.3 mg/ml mL-GDH_180_ were applied to Quantifoil R2/2 holey carbon grids and vitrified using a Vitrobot (FEI). Data collection was carried out in a Titan Krios FEI electron microscope operated at 300 kV by a K2 direct detector (GATAN) (eBIC, Electron Bio-Imaging Centre, Diamond light source, Oxford). Movie frames (1,802) were taken at a nominal magnification of ×47,170 resulting in a sampling of 1.06 Å/pixel. Each movie contained 20 frames with an accumulated dose of 40 e^−^/Å^2^. Movie frames were aligned using MotionCor2^25^, and the final average included frames 2–15 with a total dose of 28 e^−^/Å^2^ on the sample.

The contrast transfer function (CTF) of the micrographs was estimated using CTFFIND4^26^. The particles were automatically selected from the micrographs using autopicking from RELION-3^27^. Evaluation of the quality of particles and selection was performed after 2D classifications with SCIPION^28^ and RELION-3^27^ software packages. The initial volume for 3D image processing was calculated using common lines in EMAN^29^ and using the algorithm 3D-RANSAC^30^. With this initial reference, additional rounds of automated particle picking were performed. An initial data set of 276,704 particles was subjected to 2D and 3D class averaging in order to select the best particles. The 3D-classification of the 106,190 final particles with imposed D2 symmetry resulted in two different conformations, a closed (40%) and an open form (60%), with estimated resolutions of 6.6 Å and 4.47 Å, respectively. The set of particles for the open tetramer was further refined after particle polishing in RELION-3^27,31^ over dose-weighted frames (total set of 20 frames), resulting in a 3D EM map at 4.19 Å. A focused refinement on the core of the subunits (excluding blurred regions at the tip ends) further improved the resolution to 3.59 Å for a monomer in the open conformation. This refinement focused on single mL-GDH_180_ subunits was performed after the alignment of all the monomers following the D2 symmetry, with masked subunits. Local resolution was estimated using RELION-3^27,31^.

Model fitting into cryo-EM maps was performed using the programs UCSF Chimera^32^, Namdinator^33^, phenix.real_space_refine^34^ and Coot^35^. Residues 500–1588 from the crystal structure of Se-Met mL-GDH_180_ (see below) were fitted into the cryo-EM map of the open form of the protein. Se-methionine residues were replaced by methionine residues using Coot^35^ and the model was finally refined employing phenix.real_space_refine^34^ with NCS and secondary structure restraints. Overall correlation coefficients were: CC (mask): 0.73; CC (box): 0.85; CC (volume): 0.73; and CC (peaks): 0.67. The final model contained 91.6% of the residues within favored regions of the Ramachandran plot and no outliers.

Figures were generated and rendered with UCSF Chimera^32^.

Cryo-EM maps obtained for mL-GDH_180_ were deposited in the Electron Microscopy Data Bank under the accession codes EMD-11606 (open conformation), EMD-11612 (closed conformation), and EMD-11613 (monomer). Atomic coordinates for the open form of mL-GDH_180_ derived from cryo-EM data were deposited in the Protein Data Bank under the accession code 7A1D.

### Negative staining electron microscopy

Negative-stained grids of mL-GDH_180_ were prepared using 2% uranyl acetate and visualized on a JEM-1230 transmission electron microscope (JEOL Europe) at an acceleration voltage of 80 kV. Images were taken in low dose conditions at a nominal magnification of ×30,000 using a GATAN CCD camera, resulting in 2.3 Å/pixel sampling.

Labeling of N-terminally His6-tagged mL-GDH_180_ was performed by direct incubation of electron microscopy grids in solutions containing 5 nm Ni-NTA-Nanogold (Nanoprobes). Briefly, after glow discharging the grids, the protein was incubated for 1 min on the grids, fixed with 2% paraformaldehyde for 10 min at 4 °C, washed 5 min with PBS, incubated for 15 min with Nanogold diluted 1/75 in PBS, washed twice with PBS, and finally stained with 2% uranyl acetate for 45 s.

### Crystallization, X-ray data collection, and structure determination

Crystallization screenings were carried out using the sitting-drop vapor diffusion method and a Mosquito (TTP Labtech) nanoliter-dispensing crystallization robot. Crystals of Se-Met mL-GDH_180_ grew after 4–6 months from a 16.5 mg/ml protein solution containing an equimolar amount of GarA from *M. tuberculosis*, by mixing equal volumes of protein solution and mother liquor (100 mM sodium cacodylate pH 5.8, 12% v/v glycerol, and 1.25 M (NH_4_)_2_SO_4_), at 4 °C. Single crystals were cryoprotected in mother liquor containing 32% v/v glycerol and flash-frozen in liquid nitrogen. X-ray diffraction data were collected at the synchrotron beamline ID23-1 (European Synchrotron Radiation Facility, Grenoble, France), at 100 K, using wavelength 0.99187 Å. Diffraction data were processed using XDS^36^ and scaled with Aimless^37^ from the CCP4 program suite^38^.

The crystal structure of Se-Met mL-GDH_180_ was solved by molecular replacement using the program Phaser^39^. As search probe we used the atomic coordinates of a model built as follows. First, a poly-Ala model of mL-GDH_180_ was obtained from a preliminary ca. 7 Å resolution cryo-EM map of the protein, by employing the program phenix.map_to_model^40^. Features of the catalytic domain in mL-GDH_180_ monomers became apparent in the model, suggesting that the N-terminus of the polypeptide chains was located at the tips of the particle. This was confirmed by labeling N-terminally His6-tagged mL-GDH_180_ with Ni-NTA-Nanogold (Nanoprobes) and visualizing particles by negative staining electron microscopy. Then, the catalytic domain of mL-GDH_180_ (residues 702–1220) was homology-modeled by using the structure of the S-GDH_50_ from *C. glutamicum* (PDB code 5GUD) as template and employing MODELLER^41^ as implemented in the HHpred server^42^. One copy of the model of the catalytic domain was rigid-body fitted into the 7 Å cryo-EM map of mL-GDH_180_, which allowed updating the starting poly-Ala model by correcting helical elements and incorporating strands corresponding to the catalytic domain in one monomer of mL-GDH_180_. From this, the D2 tetramer was then rebuilt by applying NCS operators detected by phenix.find_ncs^43^ and the model was refined against the 7 Å cryo-EM map using phenix.real_space_refine^34^ with NCS and secondary structure restraints. Finally, one of the protein chains in the resulting model was used as search probe to solve the crystal structure of Se-Met mL-GDH_180_ by molecular replacement.

Two monomers were placed within the asymmetric unit, which taken together with nearby crystallographic symmetry mates replicate the quaternary structure observed by cryo-EM. After crystallographic refinement using phenix.refine^44,45^ with NCS and secondary structure restraints, *mFo-DFc* and *2mFo-DFc* electron density maps displayed rod-shaped electron density peaks that remained un-modeled at this stage and that most likely corresponded to helices in the N-terminal region of mL-GDH_180_. Phase improvement by density modification with RESOLVE^46^ provided additional evidence in support of such elements. The N-terminal segment of mL-GDH_180_ (residues 1-701) was modeled ab initio using RaptorX^47,48^, one of the top-ranking ab initio structure prediction methods according to recent CASP evaluations^49,50^. Raptor X works by initially estimating residue-residue contacts from residue coevolution patterns and uses the predicted contacts to drive model building; such technique has proven highly successful especially when integrated with experimental data (multiple examples overviewed in Abriata et al.^51^). The residue–residue contact map predicted by RaptorX and the models produced from it revealed that the N-terminal segment of mL-GDH_180_ comprises an array of contiguous domains, which were subsequently individually rigid-body fitted into the electron density maps. Similarly, the C-terminal domain of mL-GDH_180_ (residues 1221–1594) was modeled ab initio employing RaptorX^47,48^ and used to correct and complete the crystallographic model. Finally, un-modeled or poorly modeled segments in the CD were manually built employing Coot^35^ from a 3.59 Å resolution cryo-EM map obtained for a monomer of mL-GDH_180_. The structure was then further refined by iterative cycles of manual model building with Coot^35^, used to apply stereochemical restraints, and crystallographic refinement of atomic coordinates and individual B-factors using phenix.refine^44,45^ with NCS and secondary structure restraints. The final model contained 93% of the residues within favored regions of the Ramachandran plot and 0.2% of outliers. The crystallographic structure of Se-Met mL-GDH_180_ correctly explained the connecting loops and bulky amino acid side chains evidenced for residues 500–1588 by a 4.19-Å cryo-EM map of the protein, which allowed to validate the strategy used for model building. Furthermore, the position of Se-Met residues in the crystal structure of Se-Met mL-GDH_180_ matched the position of peaks in an anomalous difference map calculated with diffraction data acquired at 0.979338 Å (12.66 keV), the Se K-edge.

Even though Se-Met mL-GDH_180_ crystallized in the presence of GarA from *M. tuberculosis*, electron density maps did not reveal evidences of co-crystallization and molecular replacement attempts with Phaser^39^ using the atomic coordinates of GarA in PDBs 2KFU or 6I2P failed. The evidence of helical elements in all mL-GDH_180_ domains allows excluding the presence of GarA (an all beta protein) from modeled regions, particularly from those involved in crystal contacts (mL-GDH_180_ residues 1–500). The crystallization of a protein from a mixture of two or more proteins is not an unusual phenomenon, and it has even been reported that a protein can crystallize in a different space group due to the presence of other proteins in the sample without giving rise to co-crystals^52^, just to mention one example.

Figures were generated and rendered with UCSF Chimera^32^ or Pymol version 1.8.x (Schrödinger, LLC).

Atomic coordinates and structure factors obtained for Se-Met mL-GDH_180_ were deposited in the Protein Data Bank under the accession code 7JSR.

### Small angle X-ray scattering

Synchrotron SAXS data were collected at BioSAXS ID14EH3 beamline (European Synchrotron Radiation Facility, Grenoble, France) and recorded at 15 °C using a PILATUS 1 M pixel detector (DECTRIS) at a sample-detector distance of 2.43 m and a wavelength of 0.931 Å, resulting momentum transfer (*s*) ranging from 0.009 to 0.6 Å^−1^.

mL-GDH_180_ was assayed at concentrations ranging from 1 to 14 mg/ml in buffer 25 mM Tris, 150 mM NaCl, pH 7.5. For the buffer and the samples, 10 2D scattering images were acquired and processed to obtain radially averaged 1D curves of normalized intensity versus scattering angle. In order to optimize background subtraction, buffer scattering profiles recorded before and after measuring every sample were averaged. Then, for each protein sample, the contribution of the buffer was subtracted. All subsequent data processing was performed using the ATSAS suite^53^.

Average scattering curves corresponding to different protein concentrations were compared using PRIMUS^53,54^. To obtain the idealized scattering curve the low *s* region of the most diluted sample and the high *s* region of the most concentrated sample were merged. The values of the forward scattering intensity *I*(0), the radius of gyration *R*_g_ as well as the dimensionless Kratky plot were calculated using PRIMUS^53,54^. Guinier plots of independent average scattering curves evidenced a constant *R*_g_ at different protein concentrations. The Porod volume was estimated using DATPOROD^53^ and an *s*_max_ value equal to 7.5/*R*_g_. The pairwise distance distribution function *p*(*r*) and the maximum particle dimension *D*_max_ were calculated using GNOM^53,55^ with a reduced *χ*^2^ value of 1.07 for curve fitting. After running DAMMIN^53,56^ the excluded volume was estimated as *V*_ex_ = volume of a single dummy atom × number of dummy atoms/0.74). Finally, the MW was estimated from the Porod volume and the excluded volume.

### Statistics and reproducibility

One protein crystal was employed for structure determination. For EM and SAXS studies, a protein batch was prepared in each case immediately before the experiment. No data were excluded from the analyses. All attempts of replication were successful.

### Reporting summary

Further information on research design is available in the Nature Research Reporting Summary linked to this article.

## Supplementary information

Supplementary Information

Reporting Summary

## Data Availability

Cryo-EM maps obtained for mL-GDH_180_ were deposited in the Electron Microscopy Data Bank under the accession codes EMD-11606 (open conformation), EMD-11612 (closed conformation), and EMD-11613 (monomer). Atomic coordinates for the open form of mL-GDH_180_ derived from cryo-EM data were deposited in the Protein Data Bank under the accession code 7A1D. Atomic coordinates and structure factors obtained for Se-Met mL-GDH_180_ by X-ray protein crystallography were deposited in the Protein Data Bank under the accession code 7JSR.
