# Supplementary material for: 3D architecture and structural flexibility revealed in the subfamily of large glutamate dehydrogenases by a mycobacterial enzyme
Source: Commun Biol. 2021 Jun 3;4:684. doi: 10.1038/s42003-021-02222-x (PMC8175468; doi:10.1038/s42003-021-02222-x)
Supplement: Supplementary file 1 — Supplementary Information [file 42003_2021_2222_MOESM1_ESM.pdf]

**3D architecture and structural flexibility revealed in the subfamily of large glutamate dehydrogenases by a mycobacterial enzyme**

Melisa Lázaro[1], Roberto Melero[2], Charlotte Huet[3,†], Jorge P. López-Alonso[1], Sandra Delgado[1], Alexandra Dodu[1], Eduardo M. Bruch[4,†], Luciano A. Abriata[5,6], Pedro M. Alzari[3], Mikel Valle[1,*], María-Natalia Lisa[7,8,3,*]

[1] Center for Cooperative Research in Biosciences (CIC bioGUNE), Basque Research and Technology Alliance (BRTA), Bizkaia Technology Park, Building 801A, 48160 Derio, Spain.

[2] Centro Nacional de Biotecnología, CNB-CSIC, Darwin 3, 28049 Madrid, Spain.

[3] Unité de Microbiologie Structurale, Institut Pasteur, CNRS UMR 3528, Université de Paris, 25 rue du Docteur Roux, 75724 Paris, France.

[4] IGBMC, 1 Rue Laurent Fries, 67404 Illkirch, France.

[5] Laboratory for Biomolecular Modeling, School of Life Sciences, École Polytechnique Fédérale de Lausanne and Swiss Institute of Bioinformatics, Lausanne CH-1015, Switzerland.

[6] Protein Production and Structure Core Facility, School of Life Sciences, École Polytechnique Fédérale de Lausanne and Swiss Institute of Bioinformatics, Lausanne CH-1015, Switzerland.

[7] Instituto de Biología Molecular y Celular de Rosario (IBR CONICET-UNR), Ocampo y Esmeralda, Rosario S2002LRK, Argentina.

[8] Plataforma de Biología Estructural y Metabolómica (PLABEM), Ocampo y Esmeralda, Rosario S2002LRK, Argentina.

[*] Correspondence: MNL: lisa@ibr-conicet.gov.ar; MV: mvalle@cicbiogune.es

[†] Present addresses: CH, DBV-Technologies, 177-181 Avenue Pierre Brossolette, 92120 Montrouge, France; EMB, Unité de Microbiologie Structurale, Institut Pasteur, CNRS UMR 3528, Université de Paris, 25 rue du Docteur Roux, 75724 Paris, France.

## Supplementary Figure 1

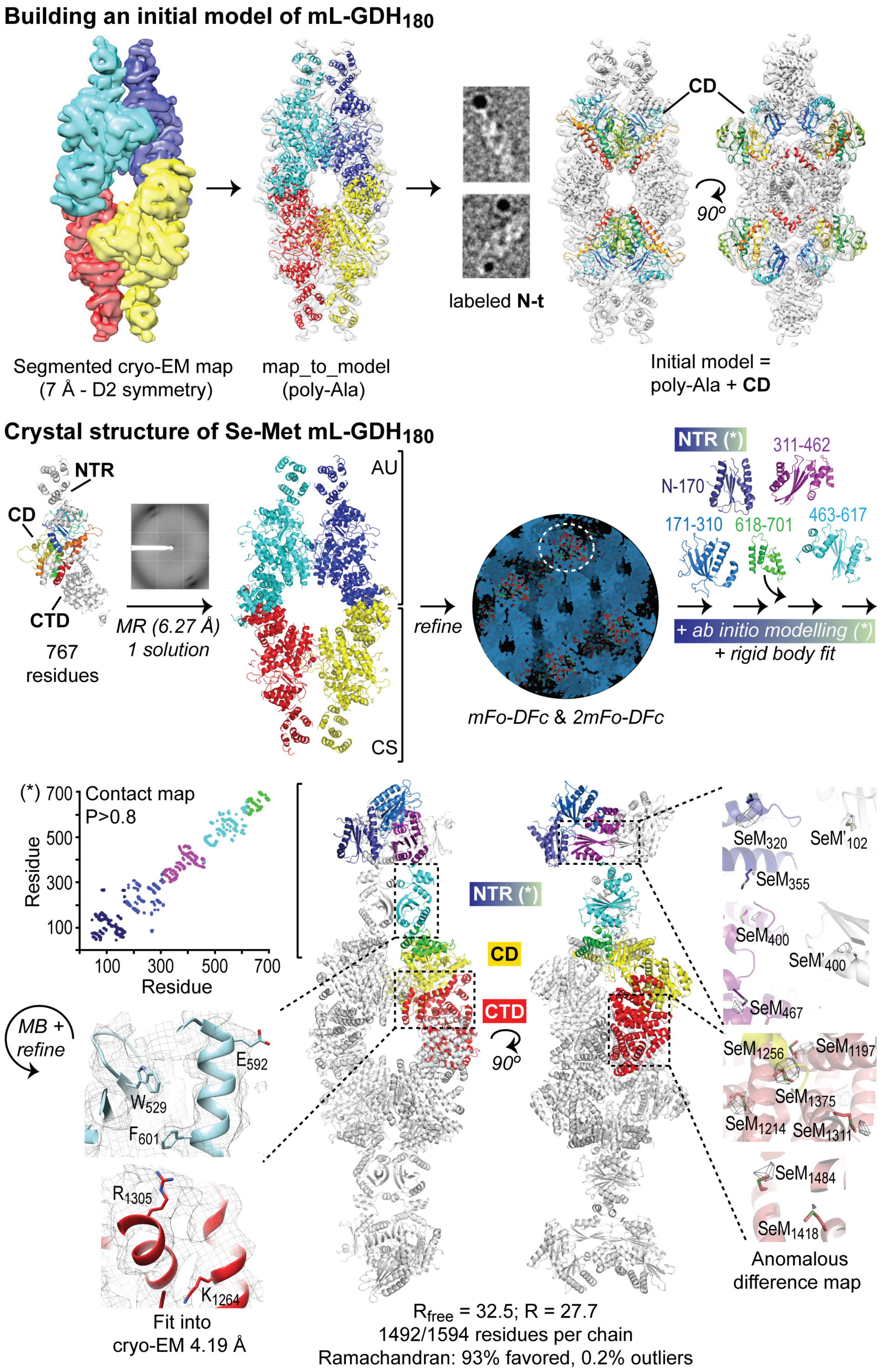

**Solving the crystal structure of Se-Met mL-GDH$_{180}$ (related to Figure 1).** Upper panel: first, from a preliminary *ca.* 7 Å resolution cryo-EM map of mL-GDH$_{180}$ (segmented rendering at the left, with subunits in different colors; semi-transparent rendering at the right), we obtained a poly-Ala model (shown as ribbons) of the protein, by employing the program phenix.map_to_model [1]. Features of the catalytic domain in mL-GDH$_{180}$ monomers became apparent in the model, suggesting that the N-terminus (N-t) of the polypeptide chains was located at the tips of the particle. This was confirmed by labeling N-terminally His6-tagged mL-GDH$_{180}$ with Ni-NTA-Nanogold and visualizing particles by negative staining electron microscopy. Then, the catalytic domain of mL-GDH$_{180}$ (CD; residues 702-1220) was homology-modeled using the structure of the S-GDH$_{50}$ from *C. glutamicum* (PDB code 5GUD) as template and employing MODELLER [2] as implemented in the HHpred server [3]. One copy of the model of the catalytic domain was rigid-body fitted into the 7 Å cryo-EM map of mL-GDH$_{180}$, which allowed updating the starting poly-Ala model by correcting helical elements and incorporating strands corresponding to the catalytic domain in one monomer of mL-GDH$_{180}$. From this, the D2 tetramer was then rebuilt by applying NCS operators detected by phenix.find_ncs [4] and the resulting model (poly-Ala + CD, with the CD in rainbow colors) was refined against the 7 Å cryo-EM map using phenix.real_space_refine [5] with NCS and secondary structure restraints. Lower panel: we used one of the protein chains in the model poly-Ala + CD (767 residues) as search probe to solve the crystal structure of Se-Met mL-GDH$_{180}$ by molecular replacement with Phaser [6]. Two monomers were placed within the asymmetric unit (AU), which taken together with nearby crystallographic symmetry (CS) mates replicate the quaternary structure observed by cryo-EM. After crystallographic refinement using phenix.refine [7,8] with NCS and secondary structure restraints, *mFo-DFc* and *2mFo-DFc* maps (in green and red contoured to 3 $\sigma$ and in blue contoured to 1.5 $\sigma$, respectively) displayed rod-shaped electron density peaks that remained un-modeled at this stage (dashed circle) and that most likely corresponded to helices in the N-terminal region of mL-GDH$_{180}$. Phase improvement by density modification with RESOLVE [9] provided additional evidence in support of such elements. The N-terminal segment of mL-GDH$_{180}$

(NTS; residues 1-701) was modeled *ab initio* using RaptorX [10,11], one of the top-ranking *ab initio* structure prediction methods according to recent CASP evaluations [12,13]. RaptorX works by initially estimating residue-residue contacts from residue coevolution patters and uses the predicted contacts to drive model building; such technique has proven highly successful especially when integrated with experimental data (multiple examples overviewed in [14]). The residue-residue contact map predicted by RaptorX ((*), showing contacts with probabilities (P) higher than 0.8) and the models produced from it (colored following the scheme of the contact map) revealed that the NTS comprises an array of contiguous domains, which were subsequently individually rigid-body fitted into the electron density maps. Similarly, the C-terminal domain of mL-GDH$_{180}$ (CTD; residues 1221-1594) was modeled *ab initio* employing RaptorX [10,11] and used to correct and complete the crystallographic model. Finally, un-modeled or poorly modeled segments in the CD were manually built employing Coot [15] from a 3.59 Å resolution cryo-EM map obtained for a monomer of mL-GDH$_{180}$ (see Figure 2C-D and Supplementary Figure 3). The structure was then further refined by iterative cycles of manual model building (MB) with Coot [15], used to apply stereochemical restraints, and crystallographic refinement of atomic coordinates and individual B-factors using phenix.refine [7,8] with NCS and secondary structure restraints. The final model contained 93% of the residues within favored regions of the Ramachandran plot and 0.2% of outliers (see also Table 1). The crystallographic structure of Se-Met mL-GDH$_{180}$ correctly explained the connecting loops (shown as ribbons) and bulky amino acid side chains (shown as sticks) evidenced for residues 500-1588 by a 4.19 Å cryo-EM map of the protein, which allowed to validate the strategy used for model building. Furthermore, the position of Se-Met residues (shown as sticks) in the crystal structure of Se-Met mL-GDH$_{180}$ matched the position of peaks in an anomalous difference map (shown as a gray mesh, contoured to 3 $\sigma$) calculated with diffraction data acquired at 0.979338 Å (12.66 keV), the Se K-edge.

## Supplementary Figure 2

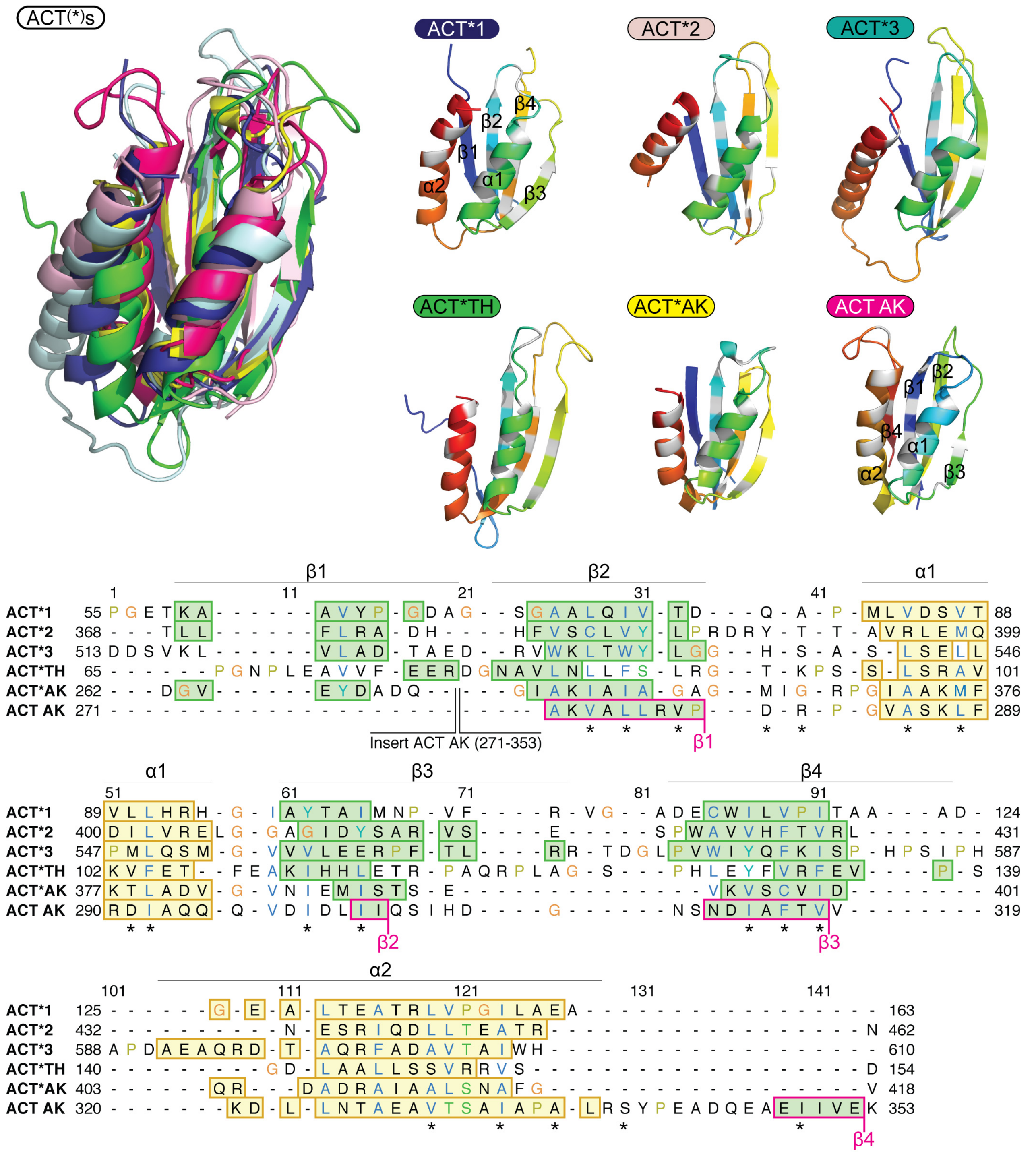


**Comparison of the ACT-like (ACT*) domains of mL-GDH$_{180}$ (related to Figure 1).** Upper panel: the ACT* domains of mL-GDH$_{180}$, tyrosine hydroxylase (TH, PDB code 2MDA) [16] and aspartate kinase (AK, PDB code 3L76) [17] as well as the ACT domain with a canonical fold of AK are superimposed (shown as ribbons). Atomic coordinates are also shown on the right in rainbow colors. The color of the labels is the same as the structures on the left. ACT* domains differ from the archetypal ACT fold in that strand β1 is located in the position usually occupied by strand β4,

creating an ACT-like ββαββα topology with a $\beta_1\beta_2\beta_4\beta_3$ antiparallel sheet. Lower panel: structure based sequence alignment of the domains in the upper panel. Secondary structure elements are displayed on the alignment. Conserved core residues previously identified in the ACT family (marked with an asterisk) are shown as white positions in the structures on the right in the upper panel.

**Supplementary Figure 3**

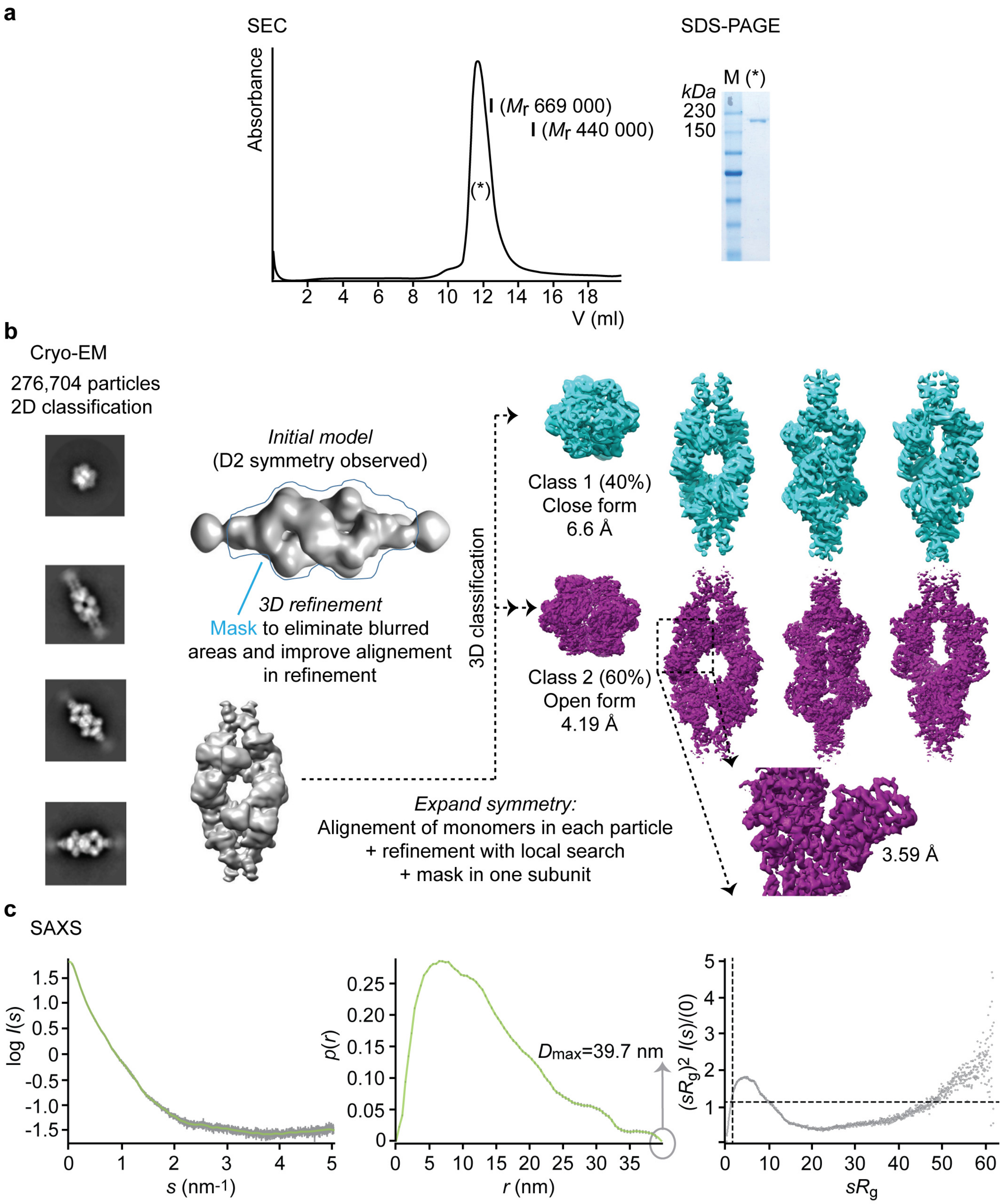


**Properties of native mL-GDH$_{180}$ (related to Figure 2). a** Left panel: a size-exclusion chromatography using a Superose 6 10/300 GL column equilibrated in buffer 20 mM MES, 300 mM NaCl, 5 mM $MgCl_2$, pH 6.0, was performed as the final step for mL-GDH$_{180}$ purification. Thyroglobulin (669 kDa) and ferritin (440 kDa) were employed as calibration standards. $M_r$:

relative molecular weight. Right panel: the purity of recombinant mL-GDH$_{180}$ (*) was evaluated by SDS-PAGE. M: molecular weight marker. Similar results were obtained for Se-Met mL-GDH$_{180}$. **b** Cryo-EM data of mL-GDH$_{180}$ was acquired and processed as detailed in Table 2. An initial data set of 276,704 particles was subjected to 2D and 3D class averaging in order to select the best particles. The 3D-classification of the 106,190 final particles with imposed D2 symmetry resulted in two different conformations, a closed (40%) and an open form (60%), with estimated resolutions of 6.6 Å and 4.47 Å, respectively. The set of particles for the open tetramer was further refined after particle polishing over dose-weighted frames (total set of 20 frames), resulting in a 3D EM map at 4.19 Å. A focused refinement on the core of the subunits (excluding blurred regions at the tip ends) further improved the resolution to 3.59 Å for a monomer in the open conformation. **c** SAXS data of mL-GDH$_{180}$ was acquired and processed as detailed in Supplementary Table 1**.** Left panel: logarithmic plot of the scattering intensity $I(s)$ (in arbitrary units) *vs.* the momentum transfer $s$, depicting the experimental data as gray dots and the fitted curve as a green line. Center panel: pairwise distance distribution function $p(r)$ (in arbitrary units) *vs.* $r$, showing the experimental data as gray dots and the fitted curve as a green line, as for the $I(s)$ *vs.* s plot, and the estimated $D_{max}$ value. Right panel: dimensionless Kratky plot. It exhibits a non-Gaussian bell shape, consistent with a properly folded protein. Besides, the position of the maximum, far from the expected for small globular proteins, and its slow decay that does not reach zero discloses an elongated protein with flexible regions. Dashed lines indicate the position were a globular protein maximum is predicted to be located ($sR_g = 3^{1/2}$ and $(sR_g)^2\, I(s)/I(0) = 1.104$) [18,19].

**Supplementary Figure 4**

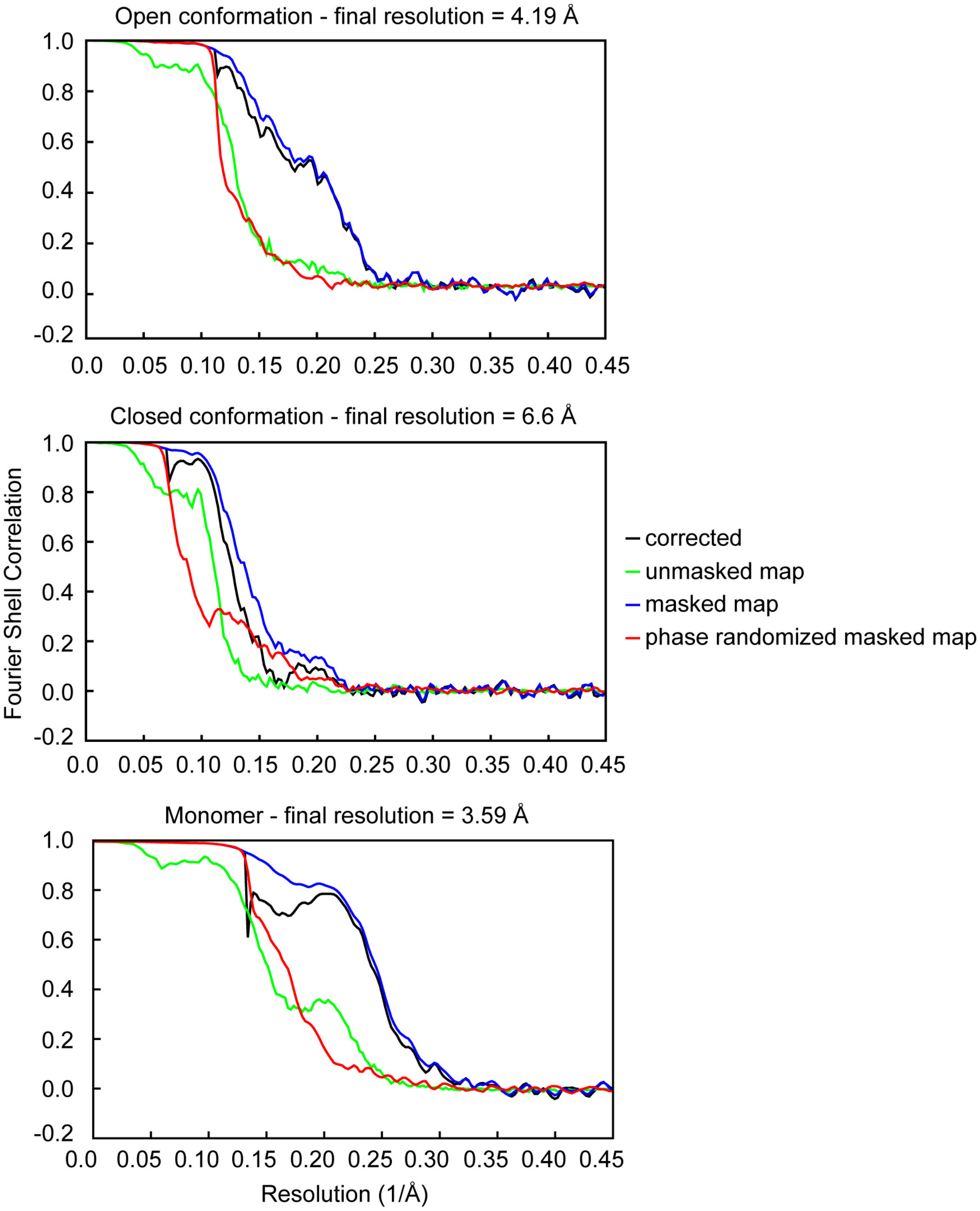


**Fourier shell correlation (FSC) for cryo-EM maps of mL-GDH$_{180}$ (related to Figure 2).** The FSC is shown between independent half-maps for the three cryo-EM maps reported. Final resolutions were estimated at the 0.14 threshold of the corresponding FSC with masked density maps.

**Supplementary Figure 5**

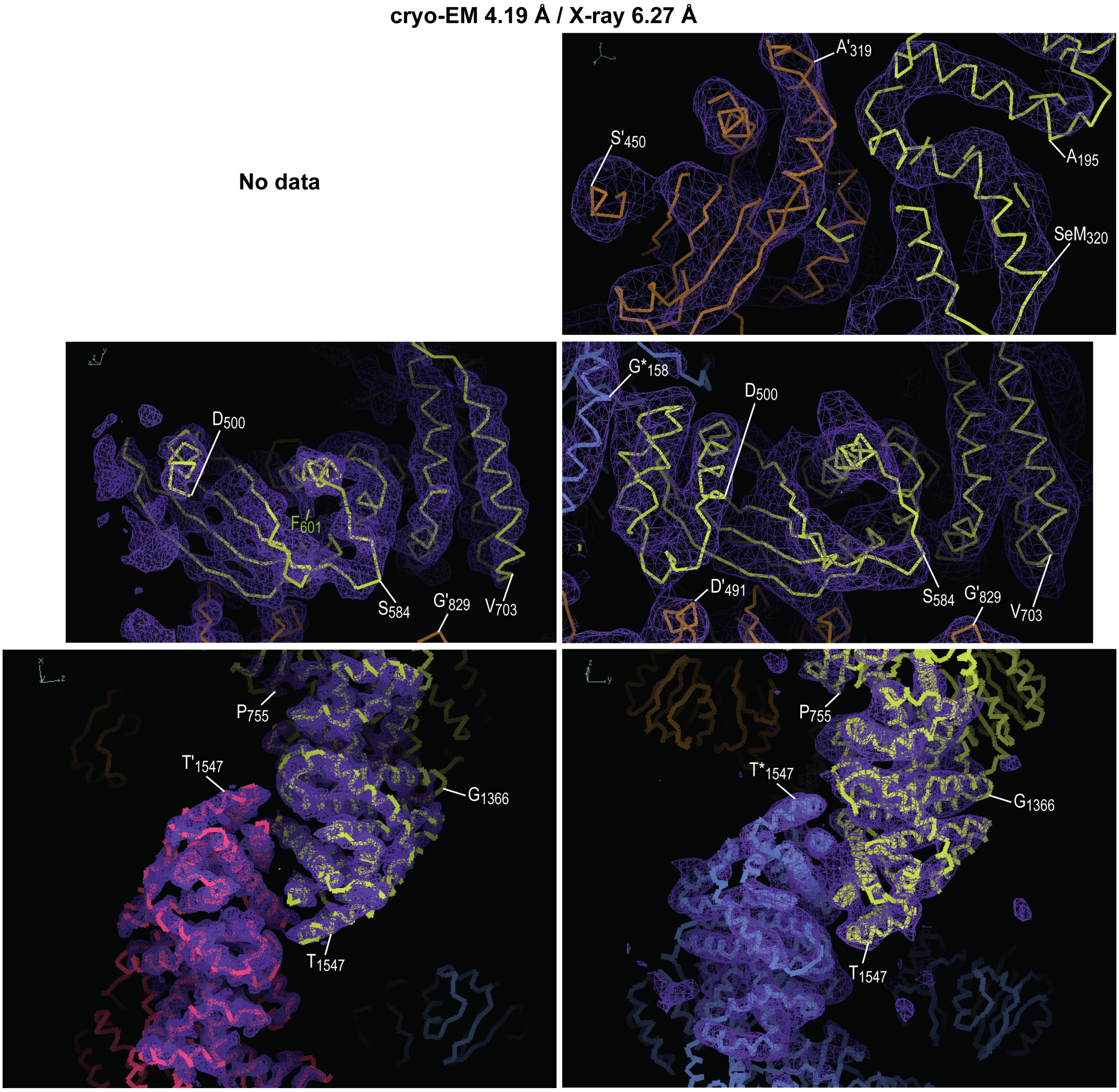


**Comparison of cryo-EM and crystallographic maps of mL-GDH$_{180}$ (related to Figures 1-2).** The upper panel shows a portion of the N-terminal region of mL-GDH$_{180}$, for which only crystallographic evidence (6.27 Å) is available. The *2mFo–DFc* crystallographic map (contoured to 1.3 σ) has comparable quality in this and other regions of the protein. The assignment of residue SeMet320 (as an example among others, see also Supplementary Figure 1) was validated by the position of Se atoms evidenced in anomalous maps. The lower panels show comparisons of the crystallographic map of mL-GDH$_{180}$ and the cryo-EM map corresponding to the open conformation of the protein (4.19 Å). Similar attributes are observed for helical elements; however, the cryo-EM

map presented better definition for beta sheets and offered additional information on amino acid side chains (for example residue Phe601, highlighted in green). Maps are displayed as blue meshes. Only the protein backbone is shown in each case. When naming amino acids, the symbols (′) and (*) denote symmetry mates and crystallographic symmetry mates, respectively.

## Supplementary Figure 6

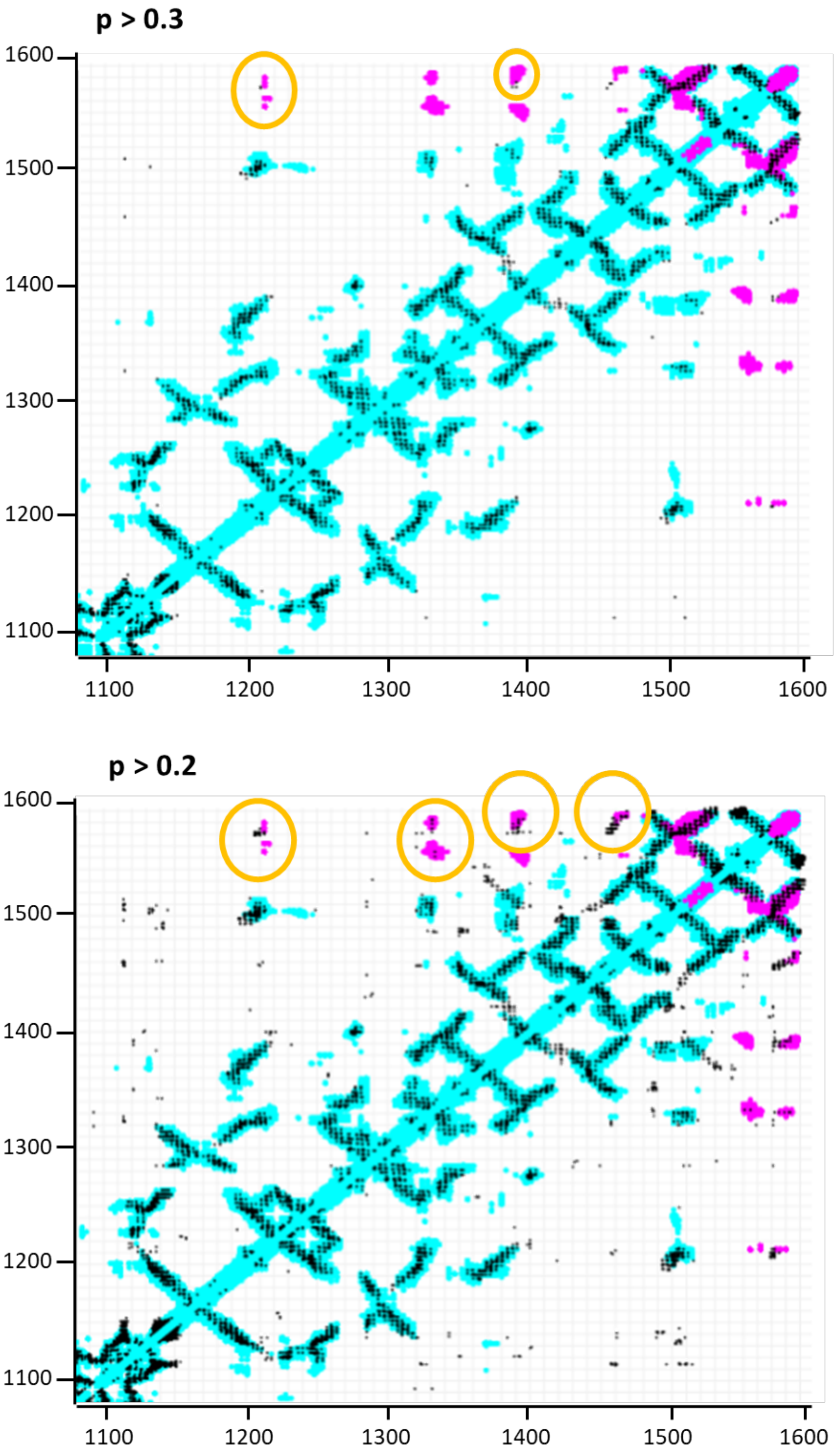


**Overlay of residue-residue contacts predicted by RaptorX for the C-terminal domain of mL-GDH$_{180}$ against the experimentally determined structure (related to Figure 1).** Cyan: intra-monomer contacts in the final experimental structure; magenta: inter-monomer contacts across

subunits interacting in the biological assembly. Black dots: contacts predicted at two P thresholds. Orange circles point at groups of predicted contacts that are only satisfied across subunits in the observed oligomer. At $p > 0.3$ predictions are dominated by intra-monomer contacts with a few lying in two regions of intersubunit contacts; at $p > 0.2$ more intersubunit contacts are evident.

**Supplementary Table 1**

**SAXS data collection and derived parameters.**

| | |
|---|---|
| **Data collection parameters** | |
| Instrument | ESRF ID14EH3 |
| Wavelength (Å) | 0.931 |
| *s* range ($Å^{-1}$)[a] | 0.009-0.6 |
| Concentration range (mg/ml) | 1.0-14.0 |
| Temperature (K) | 288 |
| **Structural parameters** | |
| *I*(0) (relative) (from p(r)) | 64.9 ± 0.3 |
| $R_g$ (Å) (from p(r)) | 106 ± 5 |
| *I*(0) (relative) (from Guinier) | 64.5 ± 0.3 |
| $R_g$ (Å) (from Guinier) | 101 ± 5 |
| $D_{max}$ (Å) | 39.7 |
| Porod volume estimate ($Å^3$) | 1200,000 ± 100,000 |
| Excluded volume estimate ($Å^3$) | 1300,000 ± 200,000 |
| Dry volume calculated [b] from sequence ($Å^3$) | 856,168 |
| **Molecular mass determination** | |
| Molecular mass $M_r$ (Da) (from Porod volume ($V_p$/1.7)) | 690,000 ± 70,000 |
| Molecular mass $M_r$ (Da) (from excluded volume ($V_{ex}$/2)) | 660,000 ± 80,000 |
| Calculated $M_r$ (tetramer, from sequence) | 705,071 |
| **Software employed** | |
| Data processing | PRIMUS [20,21] |
| Porod volume calculation | DATPOROD [20] |

[a] Momentum transfer |*s*| = 4πsin(θ)/λ

[b] Dry volume from: http://www.basic.northwestern.edu/biotools/proteincalc.html

$M_r$: molecular mass

$R_g$: radius of gyration

$D_{max}$: maximal particle dimension

$V_p$: Porod volume

$V_{ex}$: particle excluded volume

## Supplementary References


1. Terwilliger, T. C., Adams, P. D., Afonine, P. V. & Sobolev, O. V. A fully automatic method yielding initial models from high-resolution cryo-electron microscopy maps. *Nat. Methods* **15,** 905–908 (2018).
2. Sali, A. & Blundell, T. Sali, A. & Blundell, T. L. Comparative modelling by satisfaction of spatial restraints. J. Mol. Biol. 234, 779-815. *Journal of molecular biology* **234,** 779–815 (1994).
3. Zimmermann, L. *et al.* A Completely Reimplemented MPI Bioinformatics Toolkit with a New HHpred Server at its Core. *J. Mol. Biol.* **430,** 2237–2243 (2018).
4. Liebschner, D. *et al.* Macromolecular structure determination using X-rays, neutrons and electrons: Recent developments in Phenix. *Acta Crystallogr. Sect. D Struct. Biol.* **75,** 861–877 (2019).
5. Afonine, P. V. *et al.* Real-space refinement in PHENIX for cryo-EM and crystallography. *Acta Crystallogr. Sect. D Struct. Biol.* **74,** 531–544 (2018).
6. McCoy, A. J. *et al.* Phaser crystallographic software. *J. Appl. Crystallogr.* **40,** 658–674 (2007).
7. Afonine, P. V. *et al.* Towards automated crystallographic structure refinement with phenix.refine. *Acta Crystallogr. Sect. D Biol. Crystallogr.* **68,** 352–367 (2012).
8. Headd, J. J. *et al.* Use of knowledge-based restraints in phenix.refine to improve macromolecular refinement at low resolution. *Acta Crystallographica Section D: Biological Crystallography* **68,** 381–390 (2012).
9. Terwilliger, T. C. *et al.* Iterative model building, structure refinement and density modification with the PHENIX AutoBuild wizard. *Acta Crystallographica Section D: Biological Crystallography* **64,** 61–69 (2007).
10. Wang, S., Sun, S., Li, Z., Zhang, R. & Xu, J. Accurate De Novo Prediction of Protein Contact Map by Ultra-Deep Learning Model. *PLoS Comput. Biol.* **13,** e1005324 (2017).

11. Xu, J. Distance-based protein folding powered by deep learning. *Proc. Natl. Acad. Sci. U. S. A.* **116,** 16856–16865 (2019).

12. Abriata, L. A., Tamò, G. E., Monastyrskyy, B., Kryshtafovych, A. & Dal Peraro, M. Assessment of hard target modeling in CASP12 reveals an emerging role of alignment-based contact prediction methods. *Proteins: Structure, Function and Bioinformatics* **86,** 97–112 (2018).

13. Abriata, L. A., Tamò, G. E. & Dal Peraro, M. A further leap of improvement in tertiary structure prediction in CASP13 prompts new routes for future assessments. *Proteins Struct. Funct. Bioinforma.* **87,** 1100–1112 (2019).

14. Abriata, L. A. & Dal Peraro, M. State-of-the-art web services for de novo protein structure prediction. *Brief. Bioinform.* **bbaa139,** (2020).

15. Emsley, P., Lohkamp, B., Scott, W. G. & Cowtan, K. Features and development of Coot. *Acta Crystallogr. Sect. D Biol. Crystallogr.* **66,** 486–501 (2010).

16. Zhang, S., Huang, T., Ilangovan, U., Hinck, A. P. & Fitzpatrick, P. F. The solution structure of the regulatory domain of tyrosine hydroxylase. *J. Mol. Biol.* **426,** 1483–1497 (2014).

17. Lang, E. J. M., Cross, P. J., Mittelstädt, G., Jameson, G. B. & Parker, E. J. Allosteric ACTion: The varied ACT domains regulating enzymes of amino-acid metabolism. *Curr. Opin. Struct. Biol.* **29,** 102–111 (2014).

18. Doniach, S. Changes in biomolecular conformation seen by small angle X-ray scattering. *Chem. Rev.* **101,** 1763–1778 (2001).

19. Bernadó, P. Effect of interdomain dynamics on the structure determination of modular proteins by small-angle scattering. *European Biophysics Journal* **39,** 769–780 (2010).

20. Franke, D. *et al.* ATSAS 2.8: A comprehensive data analysis suite for small-angle scattering from macromolecular solutions. *J. Appl. Crystallogr.* **50,** 1212–1225 (2017).

21. Konarev, P. V., Volkov, V. V., Sokolova, A. V., Koch, M. H. J. & Svergun, D. I. PRIMUS: A Windows PC-based system for small-angle scattering data analysis. *J. Appl. Crystallogr.*

**36,** 1277–1282 (2003).
