# Supplementary material for: 3D architecture and structural flexibility revealed in the subfamily of large glutamate dehydrogenases by a mycobacterial enzyme
Source: Commun Biol. 2021 Jun 3;4:684. doi: 10.1038/s42003-021-02222-x (PMC8175468; doi:10.1038/s42003-021-02222-x)
Supplement: Supplementary file 2 — Reporting Summary [file 42003_2021_2222_MOESM2_ESM.pdf]

nature research

Corresponding author(s): Dr. María-Natalia Lisa
Dr. Mikel Valle

Last updated by author(s): Mar 27, 2021

Reporting Summary

Nature Research wishes to improve the reproducibility of the work that we publish. This form provides structure for consistency and transparency in reporting. For further information on Nature Research policies, see our Editorial Policies and the Editorial Policy Checklist.

Statistics

For all statistical analyses, confirm that the following items are present in the figure legend, table legend, main text, or Methods section.

n/a	Confirmed	
☒	☐	The exact sample size (*n*) for each experimental group/condition, given as a discrete number and unit of measurement
☒	☐	A statement on whether measurements were taken from distinct samples or whether the same sample was measured repeatedly
☒	☐	The statistical test(s) used AND whether they are one- or two-sided<br>*Only common tests should be described solely by name; describe more complex techniques in the Methods section.*
☒	☐	A description of all covariates tested
☒	☐	A description of any assumptions or corrections, such as tests of normality and adjustment for multiple comparisons
☐	☒	A full description of the statistical parameters including central tendency (e.g. means) or other basic estimates (e.g. regression coefficient) AND variation (e.g. standard deviation) or associated estimates of uncertainty (e.g. confidence intervals)
☒	☐	For null hypothesis testing, the test statistic (e.g. *F*, *t*, *r*) with confidence intervals, effect sizes, degrees of freedom and *P* value noted<br>*Give P values as exact values whenever suitable.*
☒	☐	For Bayesian analysis, information on the choice of priors and Markov chain Monte Carlo settings
☒	☐	For hierarchical and complex designs, identification of the appropriate level for tests and full reporting of outcomes
☒	☐	Estimates of effect sizes (e.g. Cohen's *d*, Pearson's *r*), indicating how they were calculated

Our web collection on statistics for biologists contains articles on many of the points above.

Software and code

Policy information about availability of computer code

Data collection

For data collection we have used the software provided with/by the following instruments/facilities:
EM:
• Titan Krios FEI electron microscope; K2 direct detector (GATAN); eBIC Electron Bio-Imaging Centre (Diamond light source, Oxford).
• JEM-1230 transmission electron microscope (JEOL Europe); CCD camera (GATAN); Electron Microscopy Platform, CICBiogune.
X-ray:
• Beamline ID23-1 (European Synchrotron Radiation Facility, Grenoble, France); PILATUS 6M_F detector (DECTRIS).
SAXS:
• BioSAXS ID14EH3 beamline (European Synchrotron Radiation Facility, Grenoble, France); PILATUS 1M pixel detector (DECTRIS).

Data analysis

EM:
• MotionCor2 (version 2_1.1.8)
• CTFFIND4 (version 4)
• RELION-3 (version 3.0.8)
• SCIPION (V2.0.0)
• EMAN (version 2)
• 3D-RANSAC (version 1)
• UCSF Chimera (1.13.1)
• Namdinator (as implemented in https://namdinator.au.dk/)
• phenix.real_space_refine (PHENIX suite version 1.14rc3-3199-000)
• Coot (version 0.8.9-pre)
X-ray:
• XDS (version November 2013)
• Aimless (CCP4 interface, version 7.0)

• Phaser (PHENIX suite version 1.14rc3-3199-000)
• phenix.map_to_model (PHENIX suite version 1.14rc3-3199-000)
• MODELLER (as implemented in https://toolkit.tuebingen.mpg.de/tools/hhpred)
• phenix.find_ncs (PHENIX suite version 1.14rc3-3199-000)
• phenix.real_space_refine (PHENIX suite version 1.14rc3-3199-000)
• Coot (version 0.8.9-pre)
• phenix.refine (PHENIX suite version 1.14rc3-3199-000)
• RESOLVE (PHENIX suite version 1.14rc3-3199-000)
• RaptorX (http://raptorx.uchicago.edu)
• Pymol (version 1.8.x)
• UCSF Chimera (version 1.12)
SAXS:
• ATSAS (including PRIMUS, DATPOROD, GNOM and DAMMIN; version 2.8.3)

For manuscripts utilizing custom algorithms or software that are central to the research but not yet described in published literature, software must be made available to editors and reviewers. We strongly encourage code deposition in a community repository (e.g. GitHub). See the Nature Research guidelines for submitting code & software for further information.

Data

Policy information about availability of data

All manuscripts must include a data availability statement. This statement should provide the following information, where applicable:

- Accession codes, unique identifiers, or web links for publicly available datasets
- A list of figures that have associated raw data
- A description of any restrictions on data availability

Cryo-EM maps obtained for mL-GDH180 were deposited in the Electron Microscopy Data Bank under the accession codes EMD-11606 (open conformation), EMD-11612 (close conformation) and EMD-11613 (monomer). Atomic coordinates for the open form of mL-GDH180 derived from cryo-EM data were deposited in the Protein Data Bank under the accession code 7A1D. Structure factors and atomic coordinates obtained for Se-Met mL-GDH180 by X-ray protein crystallography were deposited in the Protein Data Bank under the accession code 7JSR. All other data that support the findings of this study are available from the corresponding author upon reasonable request.

Field-specific reporting

Please select the one below that is the best fit for your research. If you are not sure, read the appropriate sections before making your selection.

☒ Life sciences ☐ Behavioural & social sciences ☐ Ecological, evolutionary & environmental sciences

For a reference copy of the document with all sections, see nature.com/documents/nr-reporting-summary-flat.pdf

Life sciences study design

All studies must disclose on these points even when the disclosure is negative.

Sample size	One protein crystal was employed for structure determination. For EM and SAXS studies, a protein batch was prepared in each case immediately before the experiment.
Data exclusions	No data were excluded from the analyses.
Replication	All attempts of replication were successful.
Randomization	Not relevant. A protein batch was prepared immediately before each experiment.
Blinding	Not relevant. A protein batch was prepared immediately before each experiment.

Reporting for specific materials, systems and methods

We require information from authors about some types of materials, experimental systems and methods used in many studies. Here, indicate whether each material, system or method listed is relevant to your study. If you are not sure if a list item applies to your research, read the appropriate section before selecting a response.

Materials & experimental systems

n/a	Involved in the study
☒	☐ Antibodies
☒	☐ Eukaryotic cell lines
☒	☐ Palaeontology and archaeology
☒	☐ Animals and other organisms
☒	☐ Human research participants
☒	☐ Clinical data
☒	☐ Dual use research of concern

Methods

n/a	Involved in the study
☒	☐ ChIP-seq
☒	☐ Flow cytometry
☒	☐ MRI-based neuroimaging
